# Renal mitochondrial oxidative stress is enhanced by the reduction of Sirt3 activity, in Zucker diabetic fatty rats

**DOI:** 10.1080/13510002.2018.1487174

**Published:** 2018-06-13

**Authors:** Yoshio Ogura, Munehiro Kitada, Itaru Monno, Keizo Kanasaki, Ai Watanabe, Daisuke Koya

**Affiliations:** a Department of Diabetology and Endocrinology, Kanazawa Medical University, Ishikawa, Japan; b Division of Anticipatory Molecular Food Science and Technology, Medical Research Institute, Kanazawa Medical University, Ishikawa, Japan

**Keywords:** Diabetic kidney disease, Zucker diabetic fatty rat, Sirt3, mitochondrial oxidative stress, isocitrate dehydrogenase2, superoxide dismutase2, CD38, NAD^+^/NADH ratio

## Abstract

**Objectives:** Mitochondrial oxidative stress is involved in the pathogenesis of diabetic kidney disease. The objective of our study is to identify the mechanisms of renal mitochondrial oxidative stress, focusing on Sirt3, which is nicotinamide adenine dinucleotide (NAD^+^; oxidized NAD)-dependent deacetylase in mitochondria.

**Methods:** Renal mitochondrial oxidative stress and Sirt3 activity, using Zucker diabetic fatty rats (ZDFRs) and cultured proximal tubular cells under high-glucose condition were evaluated.

**Results:** At 28 weeks of age, ZDFRs exhibited the increased urinary albumin/liver-type fatty acid-binding protein (L-FABP)/8-hydroxy-2'-deoxyguanosine (8-OHdG) excretion, histological tubular cell damage, compared to non-diabetic Zucker Lean rats. In renal mitochondria, acetylated isocitrate dehydrogenase2 (IDH2) and superoxide dismutase2 (SOD2), accompanied with mitochondrial oxidative stress and mitochondrial morphologic alterations, were increased in ZDFRs, indicating inactivation of Sirt3. Additionally, expression of the NAD-degrading enzyme, CD38, was increased, and the NAD^+^/NADH (reduced NAD) ratio was reduced in the renal cortex of ZDFRs. High-glucose stimulation in cultured proximal tubular cells also resulted in an increase in acetylated IDH2/SOD2, CD38 overexpression and a reduction in the NAD^+^/NADH ratio.

**Conclusions:** Enhancement of mitochondrial oxidative stress in the diabetic kidney was mediated by the reduction of Sirt3 activity. CD38 overexpression may be related to a reduction in the NAD^+^/NADH ratio in the diabetic kidney.

## Introduction

Diabetic kidney disease (DKD) is still a major cause of end-stage renal disease (ESRD) worldwide; therefore, a detailed mechanism for the pathogenesis of DKD should be investigated, and a novel additional therapy is necessary for the suppression of DKD. Aging is a universal process that affects all organs including kidney, and age-related disruptions in cellular homoeostasis result in decline in organ function and in the responsiveness to physiological stress. A gradual decline in renal function occurs in most healthy individuals as they age [1], and aging is recognized as one of the risk factors for the development of ESRD due to chronic kidney disease including DKD [2]. Therefore, elucidating the process of renal aging may result in a breakthrough in the treatment of DKD. Oxidative stress occurs as a consequence of reactive oxygen species (ROS) accumulation, which increases with age. In particular, mitochondria are recognized as one of the major sources of ROS, and they can also be damaged by ROS, which associates with mitochondrial dysfunction and cellular aging [3]. In the diabetic state, both overproduction of ROS from mitochondria and a reduction in the efficacy of the anti-oxidative system in mitochondria leads to the enhancement of oxidative stress in the kidney [4–6]. Thus, mitochondrial oxidative stress may be closely linked to aging and DKD.

Sirtuins are recognized as anti-aging molecules [7]. Among Sirtuins, Sirt3 is mainly located in mitochondria and plays an important role in anti-oxidative stress and cellular metabolism. A reduction in Sirt3 activity contributes to mitochondrial oxidative stress by decreasing the activation of anti-oxidative enzymes such as isocitrate dehydrogenase 2 (IDH2) and superoxide dismutase 2 (SOD2) [8–11]. In addition, Sirt3 is recognized as a reno-protective molecule [12,13]. However, whether Sirt3 dysfunction exists in the diabetic kidney and, if so, how Sirt3 dysfunction is involved in the pathogenesis of DKD are unclear. Because Sirt3 is nicotinamide adenine dinucleotide (NAD^+^; oxidized NAD)-dependent deacetylase, the levels of intracellular NAD^+^ or the NAD^+^/NADH (reduced NAD) ratio is important for the regulation of Sirt3 activity. An intracellular NAD^+^ decline is observed with aging in several tissues including the liver, heart, lung and kidney [14]. CD38 is a primary source of NAD degraded (NADase) activity and has a fundamental role as a regulator of intracellular NAD^+^ levels [15]. Camacho-Pereira et al. [16] demonstrated that CD38 is required for the age-related NAD^+^ decline and mitochondrial dysfunction through the reduction in NAD^+^-dependent Sirt3 activity in the liver, adipose tissue and skeletal muscle, which are involved in the age-related metabolic decline. However, there are no reports regarding the change in CD38 expression in the diabetic kidney. In the present study, we discovered that renal mitochondrial oxidative stress was enhanced by the reduction of Sirt3 activity, and overexpression of CD38 and a decline in the NAD^+^/NADH ratio existed in the kidney of type 2 diabetic animal model, Zucker Diabetic Fatty Rats (ZDFRs).

## Materials and methods

### Antibodies

Anti-dynamin-related protein 1 (DRP1), Cytochrome C Oxidase Subunit IV (CoxIV), β-actin, isocitrate dehydrogenase 2 (IDH2) and acetylated lysine antibodies were purchased from Cell Signaling Technology (Beverly, MA, USA). Anti-acetylated superoxide dismutase 2 (SOD2) (Lysine (Lys)-68) antibodies were obtained from Abcam (Cambridge, MA, USA). Antibodies against Sirt3, CD38 (M-19) for western blotting and CD38 (H-11) for immunohistochemistry were purchased from Santa Cruz Biotechnology (Santa Cruz, CA, USA). The anti-IDH2 (Lys-413) antibody was obtained from GeneTel Laboratories LLC (Madison, WI, USA). The anti-SOD2 antibody was purchased from Enzo Life Science (New York, NY, USA). The anti-kidney injury molecule-1 (Kim-1) antibody was obtained from R & D Systems, Inc. (Minneapolis, MN, USA).

### Experimental animals

Male Zucker Lean (fa/+) Rats (ZLRs) and male ZDFRs were provided by the Ninox Pharmaceutical Company Biological Institute (Osaka, Japan). According to manufacturer’s data, features of the ZDFRs are shown as below. The ZDFRs show the gradual increase in glucose and HbA1c levels, and body weight until 18 weeks of age, and their changes in ZDFRs reach to almost plateau at 18 weeks of age. Plasma insulin levels are increased until 8 weeks of age; however, from 10 weeks of age, the production of insulin is gradually decreased, resulting in continuous high levels of glucose and HbA1c. Although some ZDFRs turn to decrease in body weight in ZDFRs from 18 weeks of age, the degree of decrease in body weight is dependent on individual diabetic status; therefore, large differences of body weight are observed at 28 weeks aged diabetic rats. Animal studies were performed in accordance with approval by the Research Center for Animal Life Science of Kanazawa Medical University. At 28 weeks of age, individual rats were placed in metabolic cages for urine collection. After that, rats were anesthetized with isoflurane, and subsequently, the kidneys were dissected, as reported previously [17].

### Biochemical measurements

HbA1c levels were measured using a DCA 2000 Analyzer (Siemens Medical Solutions Diagnostics, Tokyo, Japan) at the end of the experiment [17]. Urinary albumin, liver-type fatty acid-binding protein (L-FABP) was measured using enzyme-linked immunosorbent assay (ELISA) kits (urinary albumin: NEPHRAT II, L-FABP: Exocell, Inc. Philadelphia, PA, USA; L-FABP: R & D Systems, Inc., Minneapolis, MN, USA) [17]. The urinary 8-hydroxy-2'-deoxyguanosine (8-OHdG) concentration was measured by ELISA kits (8-OHdG Check, Institute for the Control of Aging, Shizuoka, Japan) [5]. Urinary creatinine (Cr) was measured by a Creatinine Companion kit (Exocell, Inc., Philadelphia, PA, USA).

### Morphological analysis, immunohistochemistry and transmission electron microscopy

Paraffin sections (3 μm thick) of the kidney were stained with Masson’s Trichrome (MT) stain, and immunohistochemical staining was performed using antibodies against Kim-1 (1:100) and CD38 (1:100) [17]. Quantification of fibrosis area on MT staining, Kim-1- or CD38-positive area on immunohistochemical staining was measured using Image J software (NIH http://rsbweb.nih.gov/ij/index.html), as previously described [18,19]. Results were expressed as percentage staining per visual field (tubulo-interstitial area: ×20, glomeruli: 40×) [19]. Mitochondrial morphology in the proximal tubular cells was observed using transmission electron microscopy [17].

### Isolation of mitochondrial protein and measurement of 8-OHdG contents in mitochondrial deoxyribonucleic acid

Isolation of mitochondria from the renal cortex was performed using mitochondria extractor kits (Thermo Fisher Scientific, Rockford, IL, USA). The mitochondrial deoxyribonucleic acid (mtDNA) was extracted from the renal cortex using the mtDNA Extractor CT kit (Wako Pure Chemical Industries, Osaka, Japan). The 8-OHdG levels in DNase I-digested mtDNA were determined by ELISA using a kit (High-sensitive 8-OHdG Check, Institute for the Control of Aging, Shizuoka, Japan) [4,20].

### Western blot analysis and real-time polymerase chain reaction

Western blotting was performed using antibodies against CD38 (1:1000), β-actin (1:1000), Ace-IDH2 (1:1000), IDH2 (1:1000), Ace-SOD2 (1:1000), SOD2 (1:1000), Sirt3 (1:1000), DRP1 (1:1000), as previously described [17]. The isolation of total RNA from the renal cortex, cDNA synthesis and quantitative real-time polymerase chain reaction were performed. TaqMan probes for type 3 collagen, CD38 and Kim-1 were purchased from Thermo Fisher Scientific (Waltham, MA, USA). The analytical data were adjusted to the levels of 18s messenger RNA (mRNA) expression as an internal control, as previously described [17].

### Measurement of the NAD^+^/NADH ratio

NAD^+^ and NADH levels were measured using BioChain NAD^+^/NADH assay kits according to the manufacturer’s instructions (BioChain, Hayward, CA, USA) [16]. The principles of the assay kits are based on a glucose dehydrogenase cycling reaction, in which tetrazolium dye 3-(4,5-Dimethyl-2-thiazolyl)-2,5-diphenyltetrazolium Bromide (MTT) is reduced by NADH in the presence of phenazine methosulfate. The intensity of the reduced product color, measured at 565 nm, is proportionate to the NAD^+^ concentration in the sample. The standards attached to the kits were used to prepare the calibration curves needed to calculate the NAD^+^/NADH ratio.

### Cell culture

HK-2 cells, which are human kidney proximal tubular cells, were obtained from the American Type Culture Collection (ATCC) (Manassas, VA, USA). HK-2 cells were maintained in Dulbecco’s modified Eagle’s medium (DMEM) containing 10% fetal bovine serum and 5 mM glucose. Subconfluent HK-2 cells in 35-cm culture dishes were exposed to serum-free DMEM medium containing 5 or 30 mM glucose for 48 hours.

### Transfection of small interfering RNA

Cultured HK-2 cells were seeded in six-well plates and incubated for 24 hours. Cells were transfected with 100 nM siRNAs against Sirt3 (siRNA human SIRT3, s534084: Ambion Inc., Austin, TX, USA) or control siRNA (Negative Control #1 siRNA: Ambion Inc., Austin, TX, USA), using Lipofectamine 3000 (Invitrogen, Carlsbad, CA, USA), according to the manufacturer’s instructions. After transfection of siRNAs, cells were incubated in 10% DMEM, which is contained 5 mM glucose, for 48 hours.

### Statistical analysis

Data are expressed as the means ± standard deviation. Mann–Whitney *U* test was used for comparisons of two groups. A *p* value of <.05 was considered significant.

## Results

### Characteristics of the experimental rats

**Figure 1.**
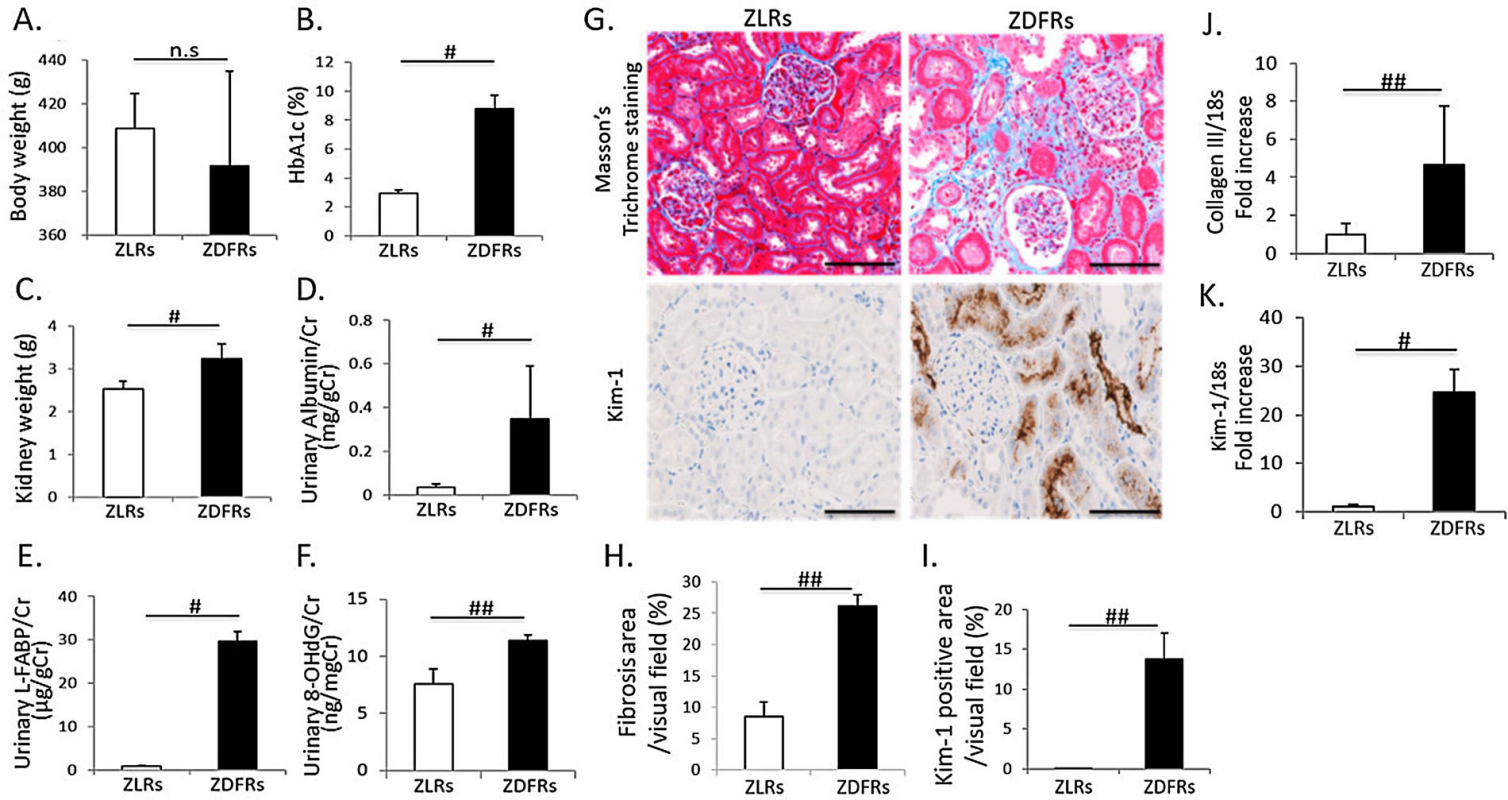
Characteristics of rats and changes in renal fibrosis and tubular cell damage at 28 weeks of age. (A) body weight, (B) levels of HbA1c, (C) kidney weight, (D) urinary albumin/creatinine (Cr) ratio, (E) liver-type free fatty acid-binding protein (L-FABP)/Cr and (F) 8-hydroxy-2'-deoxyguanosine (8-OHdG)/Cr (*n* = 6, respectively). (G) Representative photographs of Masson’s Trichrome (MT) staining (scale bar: 100 μm), immunohistochemistry for kidney injury molecule-1 (Kim-1) (scale bar: 100 μm). Quantitative data of fibrosis area (H) on MT staining and Kim-1-positive area (I) on Kim-1 immunohistochemistry per visual field of tubulo-interstitial area (MT: *n* = 6, Kim-1 IHC: *n* = 4). mRNA expression of type 3 collagen (Collagen III) (J) and Kim-1 (K), adjusted to the expression level of 18s in the renal cortex (*n* = 6, respectively). All data are mean ± SD. #*p *< .01, ##*p *< .05, n.s. denotes not significant. ZDFRs: Zucker diabetic fatty rats, ZLRs: Zucker lean rats.

Characteristics of rats at 28 weeks of age are shown in Figure 1. There was no change in body weight between non-diabetic ZLRs and type 2 diabetic ZDFRs (Figure 1(A)). However, differences of individual body weight in ZDFRs were large, as described above in the Materials and methods section. The ZDFRs exhibited significantly elevated HbA1c compared to the ZLRs (Figure 1(B)), and ZDFRs had significantly heavier kidneys than the ZFRs (Figure 1(C)). The urinary albumin/creatinine (Cr), L-FABP/Cr and 8-OHdG/Cr ratios were significantly higher in ZDFRs than in ZLRs (Figure 1(D–F)). Renal fibrosis observed using MT staining and the mRNA expression of type 3 collagen were shown to be higher in the kidneys of ZDFRs compared to the levels in the kidneys of ZLRs (Figure 1(G,H,J)). The tubular cell damage evaluated by Kim-1 immunohistochemical staining and mRNA expression was also significantly higher in ZDFRs compared to that in ZLRs (Figure 1(G,I,K)).

### Change in Sirt3 activity, oxidative stress in mitochondria and mitochondrial morphology in type 2 diabetic kidney

**Figure 2.**
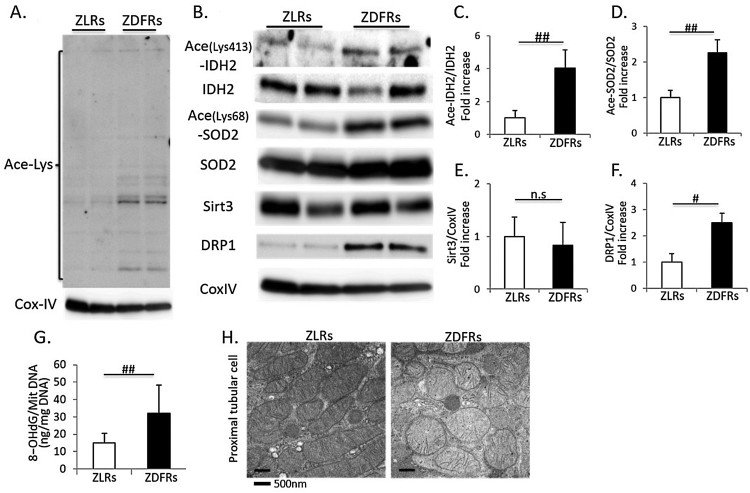
Change in Sirt3 activity, oxidative stress in mitochondria and mitochondrial morphology in the type 2 diabetic kidney. (A) Western blots of mitochondrial protein extracts from ZLRs and ZDFRs with polyclonal anti-lysine acetylation or Cytochrome C Oxidase Subunit IV (CoxIV) antibodies. (B) Representative western blots of acetylated isocitrate dehydrogenase 2 on Lys-413 (Ace-IDH2), IDH2, acetylated superoxide dismutase 2 on Lys-68 (Ace-SOD2), SOD2, Sirt3, dynamin-related protein 1 (DRP1) and CoxIV in mitochondrial protein extracts from ZLRs and ZDFRs. Quantitative ratios of Ace-IDH2 to IDH2 (C), Ace-SOD2 to SOD2 (D), Sirt3 (E) and DRP1 (F) to CoxIV (*n* = 6, respectively). (G) 8-OHdG content of mitochondrial DNA from ZLRs and ZDFRs (*n* = 6). (H) Representative transmission electron microscopy images of proximal tubular cells (scale bar: 500 nm). All data are mean ± SD. #*p *< .01, ##*p *< .05, n.s. denotes not significant. ZDFRs: Zucker diabetic fatty rats, ZLRs: Zucker lean rats.

Immunoblotting for anti-acetyl lysine-containing proteins revealed multiple bands that were far more prominent in mitochondria extracts obtained from diabetic renal cortex than in those from non-diabetic rats (Figure 2(A)). The expression levels of acetylated IDH2, SOD2 and DRP1 were significantly increased in renal mitochondria from ZDFRs compared to those from ZLRs (Figure 2(B–F)). The 8-OHdG contents in mitochondrial DNA were significantly increased in renal cortex of ZDFRs (Figure 2(G)). However, expression of Sirt3 was no different between ZDFRs and ZLRs. In addition, the mitochondrial morphology was altered in proximal tubular cells of ZDFRs; mitochondrial swelling and mitochondria without elongation were observed (Figure 2(H)).

### Change in renal expression of CD38 in type 2 diabetic rats

**Figure 3.**
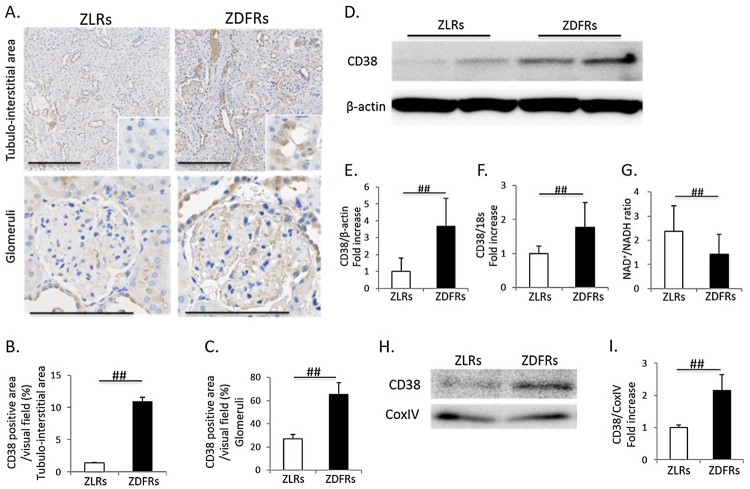
Change in renal expression of CD38 and NAD^+^/NADH ratio in type 2 diabetic rats. (A) Representative photographs of immunohistochemistry for CD38 in the tubule-interstitial area (scale bar: 200 μm) and glomeruli (scale bar: 100 μm). Quantitative data of CD38-positive area per visual field in tubulo-interstitial area (B) and in glomeruli (C) on IHC (*n* = 4, respectively). (D) Representative western blots of CD38 and β-actin in renal cortex protein extracts from ZLRs and ZDFRs. (E) Quantitative ratios of CD38 to β-actin (*n* = 6). (F) mRNA expression of CD38, adjusted to the expression level of 18s in the renal cortex (*n* = 6). (G) NAD^+^/NADH ratio in the renal cortex of ZLRs and ZDFRs (*n* = 6). (H) Representative western blots of CD38 and CoxIV in mitochondria protein extracts isolated from renal cortex of ZLRs and ZDFRs. (I) Quantitative ratios of CD38 to CoxIV (*n* = 6). All data are mean ± SD. ##*p *< .05. ZDFRs: Zucker diabetic fatty rats, ZLRs: Zucker lean rats.

CD38 is known as NADase (NAD degrade enzyme), and increases in its expression and activity may be related to an NAD^+^ decline or a reduction in the NAD^+^/NADH ratio with aging. Higher immunohistochemical staining for CD38 in both tubular cells and glomeruli was exhibited in ZDFRs (Figure 3(A–C)). The expression levels of CD38 protein and mRNA were also significantly higher in the diabetic renal cortex than in the renal cortex of non-diabetic rats (Figure 3(D–F)). The NAD^+^/NADH ratio was significantly decreased in the renal cortex in ZDFRs compared to that in ZLRs (Figure 3(G)). Additionally, the expression levels of CD38 in mitochondria protein extracts isolated from renal cortex of ZDFRs were significantly increased, compared to that of ZLRs (Figure 3(H,I)).

### Change in Sirt3 activity, CD38 expression and the NAD^+^/NADH ratio in cultured renal proximal tubular cells in a high-glucose condition

**Figure 4.**
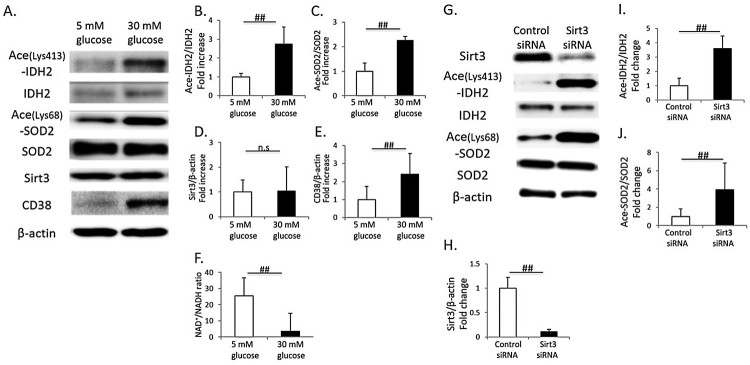
Change in Sirt3 activity, CD38 expression and the NAD^+^/NADH ratio in cultured renal proximal tubular cells (HK-2 cells) in a high-glucose condition. (A) Representative western blots of acetylated isocitrate dehydrogenase 2 on Lys-413 (Ace-IDH2), IDH2, acetylated superoxide dismutase 2 on Lys-68 (Ace-SOD2), SOD2, Sirt3, CD38 and β-actin in whole-cell lysates from cultured HK-2 cells under low glucose (5 mM) or high glucose (30 mM) conditions. Quantitative ratios of Ace-IDH2 to IDH2 (B), Ace-SOD2 to SOD2 (C), Sirt3 to β-actin (D) and CD38 to β-actin (E) (*n* = 4, respectively). (F) Intracellular NAD^+^/NADH ratio in cultured HK-2 cells under low or high glucose conditions (*n* = 4). (G) Representative western blots of Sirt3, Ace-IDH2, IDH2, Ace-SOD2, SOD2 and β-actin in whole-cell lysates from cultured Sirt3 knockdown or control HK-2 cells. Quantitative ratios of Sirt3 to β-actin (H), Ace-IDH2 to IDH2 (I), Ace-SOD2 to SOD2 (J) (*n* = 4, respectively). All data are mean ± SD. ##*p *< .05, n.s. denotes not significant.

High glucose (30 mM) induced an increase in acetylated IDH2 and SOD2 in cultured proximal tubular cells, HK-2 cells, indicating a reduction in Sirt3 activity (Figure 4(A–C)). However, Sirt3 expression was not different between HK-2 cells cultured in low or high glucose conditions (Figure 4(A,D)). In addition, the intracellular NAD^+^/NADH ratio was significantly reduced in HK-2 cells cultured under high glucose conditions, and it was accompanied with CD38 overexpression (Figure 4(A,E,F)). In Sirt3 knockdown HK-2 cells, the expression levels of acetylated IDH2 and SOD2 were significantly increased, compared to those in control HK-2 cells (Figure 4(G–J)).

## Discussion

In this study, we discovered that the reduction in Sirt3 activity was involved in renal mitochondrial oxidative stress through a decrease in the activity of anti-oxidative enzymes via increased acetylation levels of those enzymes in the kidney of type 2 diabetic animal model, ZDFRs. In addition, the expression of CD38, a NAD-degrading enzyme, was significantly increased in diabetic kidney, and it was accompanied with the intracellular NAD^+^/NADH ratio.

At first, we confirmed that ZDFRs, an animal model of type 2 diabetes, showed significant increase of HbA1c, urinary albumin and L-FABP excretion, histologically renal injuries including fibrosis and tubular cell damage, and urinary 8-OHdG excretion was also significantly elevated, which is closely related to high glucose levels. Oxidative stress plays an important role in the process of aging and age-related diseases including diabetes and DKD. Since mitochondria are a main source of ROS, the imbalance between ROS production and anti-oxidative mechanisms in mitochondria is crucial. Sirt3, 4 and 5 are located in mitochondria [21]. Although, Sirt3-5 shows deacetylase activity, the deacetylase activity of Sirt3 is much higher than that of Sirt4 and Sirt5. Sirt3 is the primary determinant of the mitochondrial acetyl-proteome [22], and the deacetylase activity is essential for the functions of Sirt3 in mitochondrial biology and pathophysiological processes including redox status. Therefore, we focused on Sirt3 activity to elucidate the mechanism of mitochondrial oxidative stress in the diabetic kidney. In this study, the levels of acetylated proteins were increased in renal mitochondria from diabetic rats, indicating that mitochondrial Sirt3 activity was reduced in the diabetic kidney. Sirt3 regulates redox status and protects mitochondria and cellular function from ROS. Someya et al. [8] reported that Sirt3 directly deacetylates and activates mitochondrial IDH2, leading to increased NADPH levels and an increased ratio of reduced-to-oxidized glutathione in mitochondria. Yu et al. [23] also demonstrated that Sirt3 activates IDH2 by deacetylating the Lys-413 residue. Our data indicated that Lys-413-acetylated IDH2 was clearly increased in the mitochondria from the diabetic renal cortex compared to those of non-diabetic rats. Additionally, SOD2 is recognized as a main anti-oxidative enzyme in mitochondria. Sirt3 regulates also SOD2 activity by deacetylating multiple lysine residues [9–11]. Qiu et al. [10] reported that calorie restriction reduces oxidative stress by Sirt3-mediated SOD2 activation through deacetylation of the Lys-68 cite on SOD2. Our data showed that acetylation of SOD2 on Lys-68 was significantly greater in renal mitochondria from diabetic rats than from non-diabetic rats. Thus, the reduction of two anti-oxidative enzymes activities in renal mitochondria of diabetic rats resulted in mitochondrial oxidative stress, which was evaluated by the 8-OHdG content in mitochondrial DNA. Both high glucose and Sirt3 knockdown also induced an increase in acetylated IDH2 and SOD2 in cultured HK-2 cells, human proximal tubular cells. In addition, alterations in mitochondrial morphology, such as mitochondrial swelling and fragmentation, were observed in diabetic proximal tubular cells, suggesting mitochondrial dysfunction. The mitochondrial DRP1 expression was significantly greater in the diabetic kidney, indicating that cellular stress, including oxidative stress, induces mitochondrial damage and fission. Previously, Morigi et al. [12] demonstrated a role for Sirt3 in reno-protection against acute kidney injury due to cisplatin-induced renal injury by using Sirt3-deficient mice, and the mechanism of Sirt3 reno-protection depended on its capacity to preserve mitochondrial integrity, thereby limiting mitochondria fission and membrane depolarization. Thus, activation of Sirt3 may be a therapeutic target for the suppression of DKD through a decrease in mitochondrial oxidative stress and maintaining of mitochondria.

Since Sirt3 is an NAD^+^-dependent deacetylase, Sirt3 activity is regulated by the intracellular levels of NAD^+^. Previous studies have suggested that tissue NAD^+^ levels decline with aging [14,24–26]. The age-related NAD decline may be caused by an increase in CD38 (NADase), which is one of the main NAD-degrading enzymes in mammalian tissue [15]. CD38 is ubiquitously expressed on the surface of the cell membrane, nucleus and mitochondria [15,27–29]. Camacho-Pereira et al. [16] demonstrated that CD38 levels increase in mouse tissues such as liver tissue, adipose tissue and skeletal muscle during aging and that CD38 is directly involved in the process that mediates the age-related NAD^+^ decline. In addition, an increase in CD38 with aging in mice was correlated with progressive mitochondrial dysfunction, which is mediated in part by the reduction in Sirt3 via the decrease in the intracellular NAD^+^/NADH ratio. In this study, we found that expression of CD38 was significantly increased in the renal cortex of diabetic rats, which is accompanied by a decrease in the NAD^+^/NADH ratio. The *in vitro* study using cultured HK-2 cells also demonstrated that high-glucose stimulation induced CD38 overexpression and reduced the intracellular NAD^+^/NADH ratio as well as the results of the *in vivo* study. Previously, Escande et al. [30] demonstrated that administration of the CD38 inhibitor, apigenin, to obese mice increased NAD^+^ levels, decreased global protein acetylation possibly through Sirt3 activation and improved several aspects of glucose and lipid homeostasis. Therefore, inhibition of CD38 may be a therapeutic target for the suppression of DKD through a restoring of Sirt3 activity via improvement of NAD^+^/NADH ratio. Moreover, in this study, the mechanism by which CD38 expression was increased in the diabetic state is unclear. Previous reports showed that inflammatory cytokines such as tumor necrosis factor-α or oxidative stress may induce overexpression of CD38 [31,32]. Further studies are necessary to address these points.

In conclusion, enhancement of mitochondrial oxidative stress in the diabetic kidney is mediated through a reduction in the activity of Sirt3. Additionally, CD38 is overexpressed in diabetic kidney, which may be associated with a decrease in intracellular NAD^+^ levels. Therefore, overexpression of CD38 in the diabetic kidney may be involved in the mitochondrial oxidative stress through the reduction of Sirt3 via a reduction in the intracellular NAD^+^/NADH ratio. However, further study is necessary to elucidate whether the inhibition of CD38 may suppress mitochondrial oxidative stress through restoring of Sirt3 activity in diabetic kidney.
